# Lung–Kidney Axis, Aging, and Cell Turnover: Current Evidence and Perspectives

**DOI:** 10.3390/cells15100875

**Published:** 2026-05-12

**Authors:** Adriana Ancer-Arellano, Yareth Gopar-Cuevas, María-de-Lourdes Chávez-Briones, Ivett Miranda-Maldonado, Sofia A. Córdova-Zúñiga, Jesús Ancer-Rodríguez, Marta Ortega-Martínez, Gilberto Jaramillo-Rangel

**Affiliations:** Department of Pathology, School of Medicine, Autonomous University of Nuevo León, Monterrey 64460, Mexico; adar7035@gmail.com (A.A.-A.); yareth.goparcu@uanl.edu.mx (Y.G.-C.); mdelchavezb@gmail.com (M.-d.-L.C.-B.); ivettmiranda77@gmail.com (I.M.-M.); azereth.cordovazng@uanl.edu.mx (S.A.C.-Z.); ancerrodriguezj@gmail.com (J.A.-R.); marta.ortegamrt@uanl.edu.mx (M.O.-M.)

**Keywords:** inter-organ communication, lung–kidney axis, aging, cell turnover, cell proliferation, apoptosis

## Abstract

Aging is the primary biological driver of progressive cellular dysfunction and a major risk factor for disease development. The lungs and kidneys are highly vulnerable to cellular damage during aging due to their continuous exposure to environmental and metabolic stressors. Increasing evidence supports the existence of a bidirectional communication axis between the lungs and kidneys. In this review, we propose an integrative mechanistic framework that links alterations in cell turnover along this axis during aging. Based on the literature reviewed, we found that age-related cellular changes induce cellular senescence. Senescent cells undergo irreversible cell cycle arrest; furthermore, telomere shortening limits cell proliferation and promotes resistance to apoptosis. However, apoptosis can increase when a critical damage threshold is reached. In this context, senescent cells acquire a senescence-associated secretory phenotype (SASP) and release circulating mediators that can transmit damage signals between the lungs and kidneys. Taken together, these processes promote a pathological feedback loop in which age-related changes in one organ can exacerbate dysfunction in another, reinforcing a bidirectional axis of damage that increases susceptibility to developing lung and kidney diseases.

## 1. Introduction

Inter-organ communication refers to bidirectional, continuous feedback between organs. This communication can be mediated by metabolites, cytokines, microRNAs, immune cells, and the senescence-associated secretory phenotype (SASP), among others, which can be transported through the circulatory or nervous systems to maintain homeostasis in different organs [1,2]. However, during aging, homeostasis is disrupted because age-induced changes in one organ can affect other organs through inter-organ communication [3].

Aging is a complex and multifactorial biological process characterized by progressive deterioration of cellular structure and function over time. This decline reduces the organism’s ability to maintain homeostasis and significantly increases its susceptibility to chronic diseases [4]. Although aging affects all tissues, the rate and severity of deterioration vary between organs [5].

The lungs and kidneys are particularly vulnerable to aging because of their continuous exposure to environmental and metabolic stressors. The lungs and kidneys have a close functional relationship mediated by the lung–kidney axis, an interdependent feedback loop between the two organs [6].

One of the mechanisms linking aging to organ dysfunction is dysregulated cell turnover. Because aging disrupts the balance between cell proliferation and apoptosis, it leads to the accumulation of senescent cells, impaired tissue renewal, and, consequently, structural remodeling of tissues. In organs such as the lungs and kidneys, where basal turnover is relatively low, even subtle alterations in this process can have significant long-term consequences [7,8,9,10]. Therefore, the aim of this review is to integrate the current evidence on alterations in cell renewal in the lung–kidney axis during aging. This will allow us to better understand the changes that occur during aging and their relationship with the development of age-related lung and kidney diseases.

For this narrative review, the PubMed and Google Scholar databases were searched, covering the period from their inception until 24 January 2026. The selected keywords were lung–kidney axis, lung–kidney crosstalk, lung–kidney axis AND aging, aging lung, aging kidney, cell turnover AND aging lung, cell turnover AND aging kidney, proliferation AND aging lung, proliferation AND aging kidney, apoptosis AND aging lung, and apoptosis AND aging kidney. Only articles published in English were included. All retrieved articles were reviewed to ensure that only those meeting the objective of this review were included. To do this, the title and abstract were examined and, when necessary, the full text of the studies was assessed to determine whether they met the inclusion criteria.

## 2. Aging

Aging involves cumulative molecular and cellular damage driven by genetic, epigenetic, metabolic, and environmental factors that influence the defense and damage repair systems. As a result, physiological functions are affected, reducing the organism’s ability to maintain homeostasis and, in turn, increasing the risk of developing various diseases [5,11,12]. The changes that occur during aging begin around the third decade of life, although clinical manifestations may appear much later [13].

One of the central mechanisms of aging is the alteration of intercellular communication, including local signaling and long-distance interactions between organs [14]. Dysfunction in inter-organ communication affects multiple systems, promoting the spread of proinflammatory and stress signals throughout the body. Furthermore, immune system dysfunction reduces the body’s ability to respond to infections and promotes a sustained proinflammatory state. As a result, a dysfunctional systemic environment is established, contributing to the progressive deterioration of organ function and the development of age-related diseases [15,16,17].

On the other hand, cell turnover is an important physiological process for maintaining tissue function and structure, consisting of a dynamic balance between cell proliferation and death [18]. Altered cell turnover can contribute to the changes observed during aging. When a cell is not replaced through cell turnover, it becomes senescent, which promotes the accumulation of damage and alteration of cell function [19]. It has also been reported that microvascular endothelial cells isolated from aged rats (20 to 24 weeks old) show decreased cell migration and proliferation, which limits angiogenesis and therefore regenerative capacity [20].

Cell turnover rates vary between tissues of the body. The total cell turnover rate in the human body has been reported to be 0.33 ± 0.02 × 10^12^ cells/day, of which, erythrocytes, neutrophils, and the epithelia of the digestive system account for 96% [21]. Although the rate of cell turnover is low in the lungs and kidneys, this process plays an important role in the changes observed during aging in these organs.

In this context, elucidating how aging-driven alterations in cell turnover impact tissue homeostasis is particularly relevant in organs such as the lung and kidney.

### 2.1. Aging Lung

Lung aging is associated with structural changes and a decline in respiratory function [22]. After birth, the lungs continue to develop until they reach peak maturity between 18 and 25 years of age; lung function remains stable until age 35, after which, it gradually declines [23].

During aging, there is a reduction in inspiratory and expiratory respiratory muscle strength, which decreases ventilation capacity [24]. In addition, the muscles experience a decrease in their cellular energy reserves [25]. In the lungs, remodeling of the extracellular matrix reduces pulmonary elastic recoil [22] due to a decrease in elastic fibers and an increase in collagen fibers [26,27]. Furthermore, the ability to clear mucus from the lungs declines [24]. Our research group also analyzed the changes that occur in the lungs during aging using a CD1 mouse model at different ages (2, 6, 12, 18, and 24 months). We observed an increase in the area of the alveoli and bronchioles [28,29], as well as a decrease in the number of alveoli and alveolar attachments in older mice [30].

Lung aging is a multifactorial process characterized by the interaction of cellular and molecular mechanisms that compromise tissue homeostasis. Type II alveolar epithelial cells develop a senescent phenotype due to oxidative stress, mitochondrial dysfunction, and DNA damage. This process is associated with activation of the p53/p21 and p16/Rb pathways, resulting in cell cycle arrest and secretion of proinflammatory factors that promote inflammation and fibrosis [31,32]. Pulmonary endothelial cells also undergo senescence, altering the integrity of the alveolar–capillary barrier. This alteration causes an increase in vascular permeability, the extravasation of inflammatory cells, inducing pulmonary edema and tissue damage [33].

The normal rate of cell turnover varies in the lung; airway cells have been reported to be renewed every 3–5 days, while alveolar cells are renewed every 4–5 weeks [34]. This turnover rate varies with age. Our research group evaluated cell turnover in lung tissue using immunohistochemistry, employing a normal aging model with CD1 mice of different ages (2, 6, 12, 18, and 24 months); the results showed an age-dependent decrease in cell proliferation, while apoptosis increased in older mice [35]. Increased apoptosis in basal and alveolar epithelial cells, as well as decreased proliferation of Clara cells, have also been reported [36]. Furthermore, aging compromises the regenerative capacity of the lung by impairing the function of type II alveolar epithelial progenitor cells and altering their niche. Changes in Wnt/β-catenin signaling, telomere shortening, and modifications of the extracellular matrix limit proliferation and differentiation into type I alveolar epithelial cells, resulting in a reduced capacity for tissue repair after injury [37]. Taken together, these alterations lead to decreased effective cell turnover and a reduced regenerative capacity, which favor the development of pulmonary dysfunction and increase susceptibility to respiratory diseases associated with aging.

### 2.2. Aging Kidney

During aging, the kidney undergoes involutive changes. These changes begin between 29 and 30 years of age, with a decrease in proximal tubular volume and GFR, which declines at an average rate of 1 mL per year [38]. From age 40 onward, the number of glomeruli progressively decreases [39]. After age 50, renal volume decreases, and the percentage of sclerotic glomeruli increases [40,41].

Other changes during aging include a decrease in the number of nephrons [42]; the presence of tubulointerstitial fibrosis due to inflammation and fibroblast activation; a decrease in tubular function caused by a decrease in the kidney’s ability to concentrate urine after water deprivation; thickening of the tubular and glomerular basement membranes due to collagen accumulation [39,43]; and an increase in glomerular basement membrane permeability due to decreased sulfation of glycosaminoglycans, which causes an increase in protein excretion [44].

The senescence of tubular epithelial cells represents a central event in renal aging. It is induced by multiple stimuli, including DNA damage, telomere shortening, oxidative stress, mitochondrial dysfunction, and endoplasmic reticulum stress, leading to irreversible cell cycle arrest and the induction of the SASP [45,46]. The accumulation of these cells limits the tubular regenerative capacity and promotes the transition from acute kidney injury (AKI) to chronic kidney disease (CKD) [45]. At the microvascular level, endothelial dysfunction and hemodynamic alterations contribute to chronic tissue hypoxia, exacerbating fibrosis and tubular cell damage. These changes are closely linked to alterations in the aging renal microenvironment [46]. Furthermore, renal aging is associated with a state of chronic low-grade inflammation induced by senescent cells’ SASP. This alteration promotes the sustained secretion of proinflammatory and pro-fibrotic cytokines, amplifying tissue damage and favoring the progression of CKD. Likewise, the activation of innate immune pathways in epithelial cells can induce tubular senescence and fibrosis [47,48].

In the adult kidney, the proliferation of glomerular and tubular cells is low, with these cells representing less than 0.5% of all proliferating cells under basal conditions [49]. However, the kidney maintains its homeostasis through the proliferation of epithelial cells and specific progenitor populations. During aging, this balance is disrupted. Antonelli et al. reported that the proliferative capacity of renal progenitor cells progressively decreases, leading to a reduction in the normal turnover of tubular cells [50]. We analyzed CD1 strain mice aged 2, 6, 12, 18, and 24 months using immunohistochemistry. We observed that with increasing age, a highly dynamic pattern of cell turnover occurs: as the number of proliferating cells increased, the number of apoptotic cells decreased, and vice versa [51]. Furthermore, it has been reported that the accumulation of senescent cells and the reduction in nephron number compromise tissue regeneration. Because the aging kidney employs compensatory mechanisms, such as polyploidization of tubular epithelial cells, to maintain function, this results in decreased cell proliferation [52]. Overall, renal aging is characterized by an imbalance between cell loss and insufficient regeneration, where reduced cell turnover and the accumulation of senescent cells contribute to the structural and functional deterioration of the kidney.

## 3. Lung–Kidney Axis

### 3.1. Physiological Interdependence

The lungs are the most important functional organs of the respiratory system because they carry out gas exchange by transferring oxygen from inhaled air to the blood [53]. The lungs are divided into the conducting zone and the gas exchange zone. The conducting zone is responsible for the transport, warming, and humidification of air, as well as the elimination of foreign particles and pathogens; it comprises the bronchi and terminal bronchioles. The gas exchange zone is responsible for transporting oxygen from the lungs to the bloodstream and for eliminating carbon dioxide from the bloodstream into the lungs; this zone comprises the respiratory bronchioles, alveolar ducts, and alveolar sacs [54].

On the other hand, the kidneys are divided into two sections, the renal cortex and the medulla [55]. Both sections contain different structures of the nephron, which are considered the functional unit. The nephron consists of the glomerulus and a complex tubular system [56]. The glomerulus and the first portion of the tubular system, called the proximal convoluted tubule, are in the renal cortex. Following this structure is the loop of Henle, which begins in the medulla and returns to the cortex to connect with the distal convoluted tubule. Finally, there are the connecting tubules, which empty into the collecting duct [57]. The kidneys perform various functions, including maintaining electrolyte homeostasis, excreting waste products, and regulating blood pressure. They also perform endocrine functions, such as the production of renin, erythropoietin, and prostaglandins, as well as the activation of vitamin D [58,59].

**Figure 1:**
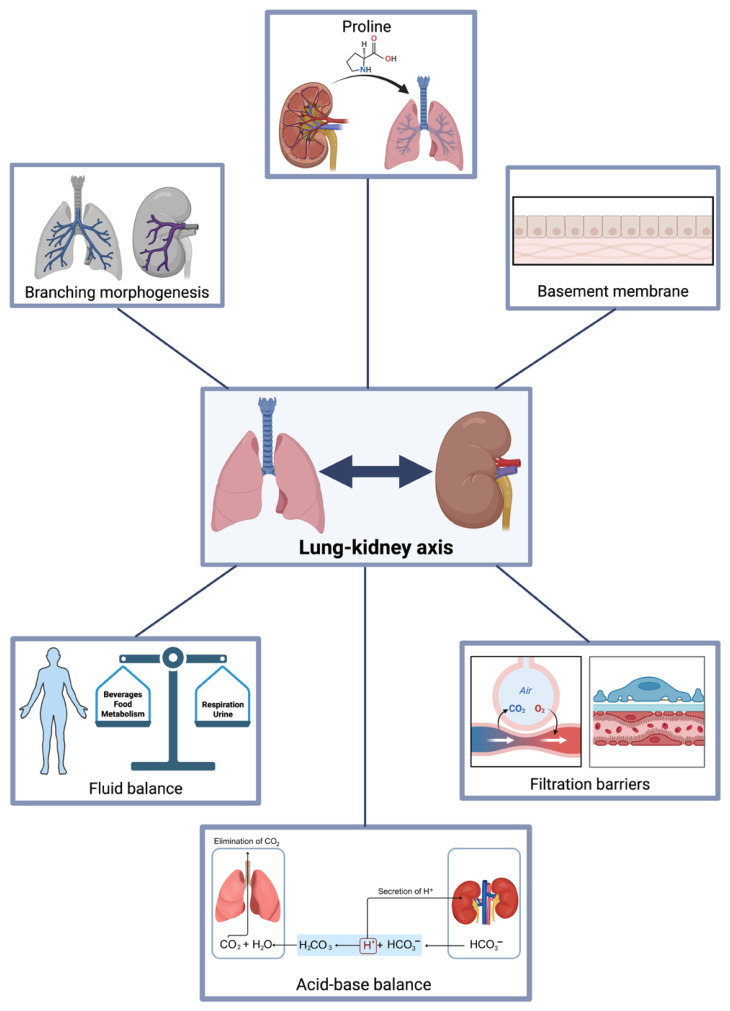
Lung–kidney axis. Schematic representation of the physiological, structural, and developmental interconnections between the lungs and kidneys. Together, these mechanisms illustrate the bidirectional nature of the lung–kidney axis.

The lungs and kidneys have related functions (Table 1). Both are highly selective filtration barriers: the lungs exchange gases and the kidneys filter blood [60]. They are the main organs that regulate the acid–base balance in humans. The lungs regulate blood pH by adjusting CO_2_ levels via respiration. The kidneys regulate pH by excreting hydrogen ions, and reabsorbing and producing bicarbonate [61]. Both organs are also involved in regulating fluid balance. The lungs affect fluid balance by losing water through respiration, while the kidneys maintain fluid balance by filtering the blood, reabsorbing essential elements, and excreting excess water and electrolytes (Figure 1) [6,61].

Furthermore, both organs share similarities in the composition of their basement membrane (BM). The BM is an extracellular structure composed of connective tissue that functions as an anatomical barrier [62]. Its general composition is uniform across all tissues, consisting of type IV collagen, laminins, and proteoglycans. The BMs of the lung and kidney share similarities in certain isoforms of type IV collagen, particularly those that contain heterodimer chains formed by the α3, α4, and α5 subunits [63,64,65]. The BM facilitates cell-to-cell communication through its components. Nearby inflammatory cells, whether resident or infiltrated, participate in BM turnover by secreting proteases. In this turnover process, biologically active fragments of the BM are released, which may have functions different from those of their progenitor molecules, such as cell signaling. Given the similarities between the basement membranes of the lung and kidney, these biologically active BM fragments may be involved in communication along the lung–kidney axis (Figure 1) [66].

Interestingly, during embryonic development, the lungs and kidneys form through the same process, called branching morphogenesis. This process involves remodeling of the epithelial sheaths, which branch into multicellular tubular networks in the surrounding mesenchyme to form a complex epithelial tree, thereby maximizing the functional area within a limited 3D space [67,68]. During this process, the kidneys and lungs generate highly arborized epithelial networks from bud primordia, but the mechanisms differ between the two organs. In the lungs, Nkx2.1-positive epithelial cells in the ventral foregut initiate branching through lateral branching, and planar and orthogonal bifurcation [69,70]. In contrast, in the kidneys, the ureteric bud undergoes repeated tip bifurcations, followed by some trifurcations (Figure 1) [71].

The direct interaction between the lung and kidney begins during gestation. In the first trimester of pregnancy, the kidneys are the main source of proline, which is necessary for collagen synthesis and, therefore, for the maturation of the lung parenchyma [72,73]. In the last stage of pregnancy, the urine produced by the kidneys is a fundamental component of the amniotic fluid, which helps in the maturation and growth of the lungs by increasing the hydrostatic pressure, thereby expanding the airways (Figure 1) [74].

Taken together, these structural, functional, and developmental connections highlight a close physiological relationship between the lungs and kidneys, which can be affected by various factors, including aging.

### 3.2. Lung–Kidney Axis: Mechanisms of Aging and Their Relationship with the Development of Diseases

#### 3.2.1. Oxidative Stress

Reactive oxygen species (ROS) are produced during various cellular processes, including cell metabolism and responses to foreign organisms. Under physiological conditions, ROS function as signaling molecules that regulate basic biological processes such as cell proliferation, differentiation, immune response, and inter-organ crosstalk [75,76]. Oxidative stress (OS) occurs when there is an imbalance between ROS production and antioxidant defense enzymes, leading to ROS accumulation and damage to lipids, proteins, and DNA [77].

In the lung, oxidative stress is a central feature of multiple age-related pathologies. In patients with acute respiratory distress syndrome (ARDS), oxidative stress has been detected, with increased ROS generation in pulmonary endothelial cells and an increase in oxidized proteins, as well as decreased glutathione levels [78,79,80,81]. Patients with COPD also exhibit oxidative stress because they have a deficiency in NRF2, a key transcription factor that regulates the antioxidant response [82]. In patients with idiopathic pulmonary fibrosis, their pulmonary fibroblasts have been found to have deficiencies in antioxidant markers, including superoxide dismutase 1, heme oxygenase, glutathione reductase, and NRF2, promoting a profibrotic redox environment [83,84].

In the kidney, oxidative stress is closely related to mitochondrial dysfunction. In patients with CKD, the electron transport chain is altered, leading to increased ROS and mitochondrial membrane damage, which induces apoptosis [78,85].

#### 3.2.2. Cellular Senescence

Cellular senescence is a permanent state of cell cycle arrest [86]. Several factors promote cellular senescence, including telomere shortening, DNA damage, epigenetic modifications, mitochondrial dysfunction, loss of tumor suppressors, and oxidative stress [87]. This process is primarily regulated through tumor suppressor pathways involving p16, p21, and p53 [88,89,90].

When a cell enters senescence, it remains metabolically active and undergoes changes in the expression and secretion of proteins, including proinflammatory cytokines, chemokines, matrix metalloproteinases, growth factors, and damage-associated molecular patterns (DAMPs). These factors (which characterize the SASP) can enter the systemic circulation, providing a mechanistic basis for inter-organ communication through soluble mediators that propagate inflammation and senescence in organs [91].

The pattern of protein secretion (SASP) is specific to each cell type and is involved in inflammaging [92]. Inflammaging is the activation of the innate immune system in the absence of an immunological threat. This process is characterized by elevated levels of tissue and circulating proinflammatory cytokines, specifically IL-1β, IL-6, and tumor necrosis factor-α (TNF-α) [93]. The presence of a low degree of chronic inflammation and cellular senescence forms a vicious cycle due to autocrine and paracrine cell communication, which can cause lung progenitor cells to lose their ability to repair damaged tissues [94].

Tsuji et al. reported that the lungs of COPD patients had a higher percentage of proinflammatory senescent cells expressing p16(INK4a) and phosphorylated NF-κB compared with the tissue of smokers and asymptomatic non-smokers [95]. Another study evaluated the supernatants of fibroblast cultures derived from COPD patients and found that the COPD-associated SASP protein profile includes the cytokines IL12B, TNFSF14, and RANKL, and the chemokines CCL15, CCL23, and CXCL9, which may play a role in the chronic inflammatory response observed in COPD [96].

Studies in animal models of AKI have identified, during the early stages of the disease, p16- and p21-positive senescent renal tubular epithelial cells, which are associated with the development of interstitial fibrosis via SASP-mediated fibroblast proliferation and extracellular matrix protein deposition; an increase in the secretion of proinflammatory cytokines such as IL-6 and IL-8 has also been observed [45,48,97]. Chkhotua et al. evaluated the expression of the cyclin-dependent kinase inhibitors p16 and p27 in tissue from healthy human subjects throughout aging. They analyzed healthy kidney tissue from subjects aged 21 to 80 years and observed that p16 expression in cortical and interstitial tubular cells increased with age, whereas p27 only increased in cortical tubular cells with age [98].

#### 3.2.3. Cell Turnover Imbalance and Disease

Cell turnover has also been studied in relation to the development of age-related lung diseases. Reports indicate that apoptosis plays a significant role in the development of pulmonary fibrosis. During the development of this disease, there is an increase in apoptosis in epithelial cells, which prevents re-epithelialization of lung tissue and impairs its function. The presence of apoptosis-resistant fibroblasts also promotes fibrosis; furthermore, there is a decrease in the clearance of apoptotic cells by granulocytes, thereby maintaining a persistent inflammatory state [99]. Calabrese et al. evaluated cell renewal in patients with pulmonary emphysema undergoing transplantation and found that apoptosis was increased in alveolar cells compared with the control group; interestingly, no difference in cell proliferation was observed [100].

It has been reported that increased apoptosis due to BCL-2 deficiency promotes the development of cystic kidney disease [101]. On the other hand, Schmitt et al. evaluated the effect of aging on cell proliferation in a murine model of ischemia–reperfusion-induced AKI. The kidneys of young mice (2–3 months old) showed significantly higher proliferation rates in the outer medulla (OM) compared with older mice (19–24 months old) [102]. Furthermore, Cardiani et al. evaluated cell turnover in the proximal tubule epithelium of rats aged 4, 6, 10, and 23 months. They observed that cell proliferation increased in 6- and 10-month-old mice and subsequently decreased in 23-month-old mice; the 10-month-old group showed the highest proliferation rate among the analyzed groups. Interestingly, no changes in apoptosis were observed [103].

Taken together, these mechanisms (oxidative stress, cellular senescence, and impaired cell turnover) are not independent processes but interconnected pathways that reinforce each other during aging. Their integration provides a mechanistic framework that explains how aging promotes the development and progression of lung and kidney diseases.

### 3.3. Clinical Implications of Bidirectional Communication of the Lung–Kidney Axis

Inter-organ communication between the lung–kidney axis is evident in the development of diseases that affect both organs (Table 2).

Previously published studies indicate that AKI can trigger a systemic response capable of affecting the lungs. Renal ischemia induces the release of circulating inflammatory mediators, including TNF-α, which activate TNFR1-dependent signaling in lung tissue, promoting caspase activation and, consequently, pulmonary endothelial cell apoptosis. This process is accompanied by local inflammatory activation characterized by increased production of cytokines and adhesion molecules, which favors the recruitment of immune cells [104,105]. Furthermore, it has been reported that T lymphocytes activated in the kidney can migrate to the lungs, leading to an increased inflammatory response that contributes to tissue damage and the induction of apoptosis [106]. This coordinated interaction between TNF-dependent signaling, immune activation, and endothelial apoptosis disrupts the balance between cell death and tissue repair, contributing to dysregulation of cell turnover and the progression of multiple organ dysfunction.

**Table 2:** Diseases related to lung–kidney axis dysregulation.

Renal Disease	Pulmonary Disease	Mechanisms	Inter-Organ Effects	Key Factors	References
AKI	ARDS	Hydrostatic edema: reduced urine output and reduced cardiac output promote fluid overload. Non-hydrostatic edema: alveolocapillary barrier disruption due to uremia, systemic inflammation, apoptosis, and oxidative stress.	Kidney injury promotes lung injury via systemic inflammation and endothelial dysfunction.	Uremia, cytokines, oxidative stress, and apoptosis.	[107,108]
CKD	COPD	Chronic inflammation, oxidative stress, and endothelial dysfunction.	COPD → CKD: hypoxia and hypercapnia induce renal vasoconstriction and reduce renal blood flow. CKD → COPD: fluid overload leads to pulmonary edema and respiratory muscle dysfunction.	Smoking, systemic inflammation, and metabolic and endocrine alterations.	[109,110,111,112]
CKD	Pulmonary hypertension	Inflammation causes vascular calcification and stiffening. Uremic toxins impair vasoregulation. Alterations in calcium/phosphate metabolism increase vascular calcification and pulmonary vascular resistance.	CKD-driven vascular remodeling impacts pulmonary circulation.	Uremic toxins, mineral metabolism disorders, and chronic inflammation.	[109,113,114,115]
CKD	Idiopathic pulmonary fibrosis	Oxidative stress leads to increased ROS production. These species activate TGF-β, which induces profibrotic signaling and immune dysregulation.	Systemic pro-fibrotic environment links kidney dysfunction with lung fibrosis.	ROS, TGF-β signaling, and chronic inflammation.	[109,116]

AKI, acute kidney injury; ARDS, acute respiratory distress syndrome; CKD, chronic kidney disease; COPD, chronic obstructive pulmonary disease; ROS, reactive oxygen species.

Patients with AKI often develop ARDS as a complication. This complication develops due to the formation of pulmonary edema through hydrostatic and non-hydrostatic mechanisms [107]. Hydrostatic edema occurs due to decreased urine and cardiac output. Non-hydrostatic edema disrupts the alveolocapillary barrier due to uremia, systemic inflammation, apoptosis, and increased oxidative stress, leading to fluid accumulation in the lungs [108].

Patients with CKD have a higher prevalence of chronic obstructive pulmonary disease (COPD). There are risk factors common to both diseases, such as advanced age, smoking, and high levels of inflammatory markers [109]. Tobacco smoke contains compounds, such as nicotine and heavy metals, that promote the development of these diseases. Nicotine increases oxidative stress and raises blood pressure by activating the sympathetic nervous system [110]. Patients with CKD exhibit a decreased glomerular filtration rate (GFR), leading to pulmonary edema and respiratory muscle dysfunction due to fluid retention and metabolic, endocrine, and cardiovascular changes in the muscles [111]. Numerous studies showed an association between COPD and CKD, and it is not always possible to distinguish which is the primary disease [109]. Patients with COPD may develop CKD as the presence of hypercapnia and hypoxia can alter perfusion by activating vasoactive systems, inducing renal vasoconstriction, and thereby reducing renal blood flow [112].

Furthermore, patients with CKD have a higher prevalence of pulmonary hypertension and idiopathic pulmonary fibrosis [109]. Pulmonary hypertension develops due to inflammation, which causes vascular hardening and calcification [113]; uremic toxins, which alter vasoregulation, leading to direct dysfunction of the pulmonary circulation [114]; and alterations in calcium and phosphorus metabolism can also increase the risk of developing vascular calcification and increased pulmonary resistance [115]. Idiopathic pulmonary fibrosis develops due to inflammation and oxidative stress. Excess reactive oxygen species activate the profibrotic cytokine transforming growth factor-β (TGF-β), which leads to dysregulation of the immune system and increased fibrotic responses [116].

Aging is a significant risk factor for the development of the aforementioned diseases [109,117]. Therefore, studying the changes in the lungs and kidneys that occur with aging is critical to improving our understanding of the mechanisms underlying these diseases.

## 4. Conclusions

**Figure 2:**
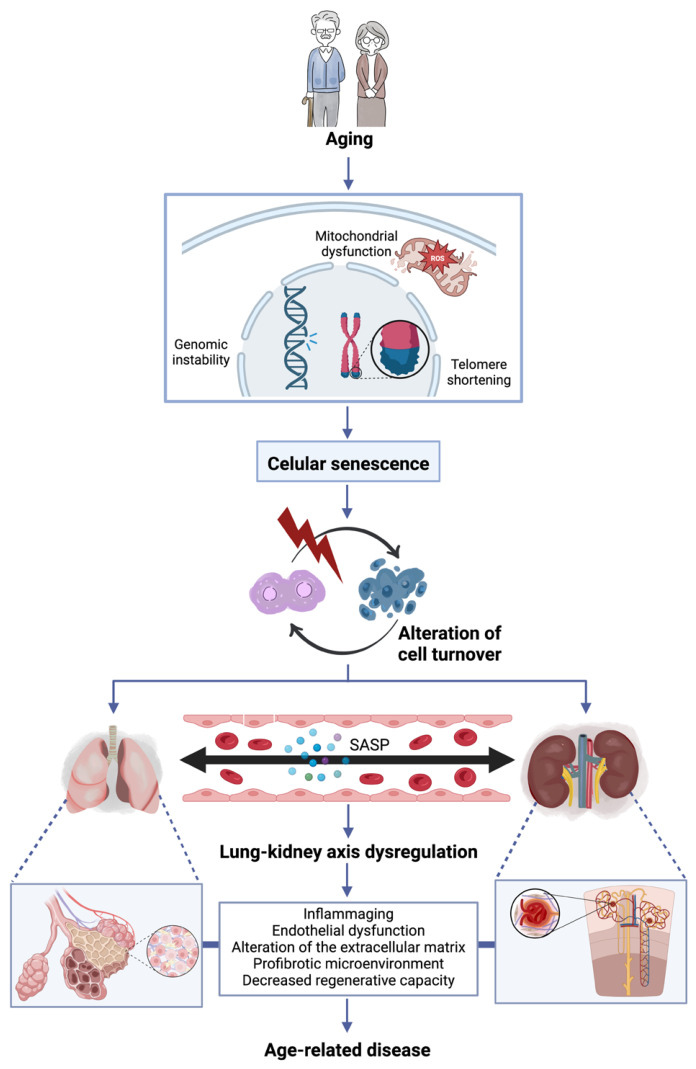
Lung–kidney axis dysregulation. The age-dependent accumulation of senescent cells disrupts tissue homeostasis by impairing cell turnover. Senescent cells acquire a senescence-associated secretory phenotype (SASP), which promotes inflammaging and endothelial dysfunction. These alterations establish a pathological feedback loop in which damage signals propagate bidirectionally between the lung and kidney, promoting fibrosis and decreasing regenerative capacity, thus increasing susceptibility to age-related diseases.

Based on the literature reviewed, we can conclude that cellular senescence is a key feature of lung and kidney aging, disrupting tissue homeostasis by affecting cell turnover. This occurs because senescent cells undergo irreversible cell-cycle arrest in response to cumulative effects from cellular stressors, such as oxidative stress. Furthermore, telomere shortening limits cell proliferation and promotes resistance to apoptosis. However, apoptosis can increase when a critical damage threshold is reached. Taken together, the age-dependent accumulation of senescent cells and the decline in stem and progenitor cell function impair tissue repair and, consequently, lead to a progressive loss of tissue structural and functional integrity. In this context, systemic factors cause damage to the lung–kidney axis. Persistent inflammation and the SASP promote the release of circulating mediators that can cause endothelial dysfunction. These interconnected mechanisms establish a pathological feedback loop in which damage signals propagate bidirectionally between the two organs, promoting fibrosis and decreased regenerative capacity. This integrated systemic dysregulation underlies the coordinated decline in lung and kidney function, increasing susceptibility to age-related diseases in both organs (Figure 2).

Understanding how aging alters communication between the lung and kidney is essential for developing integrative therapeutic strategies that target systemic mechanisms rather than organ-specific ones.

## 5. Perspectives

Inter-organ communication has emerged as a key field for understanding systemic physiology and disease. The relevance of inter-organ communication in aging has been formally recognized as a priority research area, with dedicated institutional support and funding, highlighting its potential role in regulating longevity [2].

Previous reports indicate that blood contains thousands of secreted proteins whose roles in organ signaling have not been fully characterized, revealing a significant gap in our understanding of these mediators. Since large-scale, systematic in vivo studies are limited in mammals, genetic models such as Drosophila have been fundamental in advancing the identification of mechanisms of inter-organ communication [118]. In recent years, this field of research has experienced significant growth thanks to advances in multi-omics, artificial intelligence, and imaging technologies. Large-scale population studies, such as those conducted at the UK Biobank, have enabled the mapping of complex interactions between organs, for example, in the heart–brain–liver axis, helping to identify new metabolic connectivity networks between these organs [1]. Furthermore, the development of multi-organ-on-a-chip (multi-OoC) platforms represents a key technological advance, enabling integrated modeling of multiple organs under controlled conditions. These systems overcome the limitations of single-organ models and have the potential to revolutionize biomedical research and facilitate the study of multisystemic diseases. These tools are transforming our ability to unravel complex physiological processes and are opening new opportunities for medical innovation [119]. However, they still face technical challenges for widespread application.

It is important to mention that, although age-related alterations in cell turnover have been identified in the lungs and kidneys, integrated studies of the lung–kidney axis remain limited.

Further research in this area is essential for several reasons. First, many age-related lung and kidney diseases do not occur in isolation but rather as part of a systemic syndrome where inter-organ communication plays a central role. Furthermore, the mechanisms linking alterations in cell turnover during aging across both organs have not been fully characterized. Second, most studies have focused on single-organ models. More integrative experimental approaches, such as those described above, are needed to capture the dynamics of cell turnover within a systemic context. However, without a detailed characterization of the shared and specific mechanisms, these approaches remain largely associative descriptions. Finally, the increase in life expectancy globally makes this type of research even more urgent. Population aging is associated with a greater burden of chronic multi-organ diseases, underscoring the need to understand not only the cellular changes intrinsic to each organ, but also the communication networks that interconnect them.

Overall, advancing research on cell renewal in the lung–kidney axis during aging will not only help us better understand the aging mechanisms underlying the dysfunction of these organs, but also aid in the identification of new therapeutic targets and the design of strategies to prevent and treat age-related chronic lung and kidney diseases.

## Figures and Tables

**Table 1:** Physiological interdependence between the lung and kidney.

Mechanism	Source Organ	Target Organ	Mediators	Physiological Function	Reference(s)
Systemic oxygenation	Lung	Kidney	Arterial O_2_	Maintenance of renal perfusion	[53]
Acid–base regulation	Lung	Kidney	CO_2_	Control of pH	[61]
Acid–base regulation	Kidney	Lung	HCO_3_^−^ (reabsorption), H^+^ (secretion)	Control of pH	[61]
Fluid balance	Kidney	Lung	Sodium and water	Prevention of pulmonary edema	[6,61]

O_2_, oxygen; CO_2_, carbon dioxide; HCO_3_^−^, bicarbonate; H^+^, hydrogen ions.

## Data Availability

No new data were created or analyzed in this study.

## Abbreviations

The following abbreviations are used in this manuscript:

AKI	Acute kidney injury
ARDS	Acute respiratory distress syndrome
BM	Basement membrane
CO_2_	Carbon dioxide
COPD	Chronic obstructive pulmonary disease
DAMPs	Damage-associated molecular patterns
GFR	Glomerular filtration rate
mL	Milliliters
OS	Oxidative stress
pH	Potential of hydrogen
ROS	Reactive oxygen species
SASP	Senescence-associated secretory phenotype
TGF-β	Transforming growth factor-β
TNF-α	Tumor necrosis factor-α
